# Social Determinants of Health and Related Inequalities: Confusion and Implications

**DOI:** 10.3389/fpubh.2019.00011

**Published:** 2019-02-08

**Authors:** M. Mofizul Islam

**Affiliations:** Department of Public Health, La Trobe University, Melbourne, VIC, Australia

**Keywords:** social determinant of health, public health policies in health, health inequalites, social health disparities, social health

In recent decades, “social determinants of health” has received considerable attention as a foundational concept in the field of population and public health (1). An online search using the term “social determinants of health” retrieves numerous articles and documents, most of which have been published in recent years. The work of the World Health Organization's Global Commission on Social Determinants of Health played an important role in drawing attention to the concept of social determinants of health, as did the World Conference on Social Determinants of Health in Brazil in 2011. Because the social determinants of health demand a multifaceted perspective, and that there has been a rapid emergence of theoretical models and frameworks and rise in the volume of literature in a relatively small period, substantial ambiguities exist around this concept (2). These ambiguities deter in communicating the key message of “social determinants of health” to the major stakeholders such as healthcare providers, policy-makers, researchers, general public or students. Given that social determinants of health are vital for overall public health achievement, a clear understanding of the concept is crucial. This paper attempts to discuss some of the ambiguities and or confusions surrounding the concept.

The World Health Organization defines social determinants of health as conditions or circumstances in which people are born, grow, live, work, and age. These conditions are shaped by political, social, and economic forces (3). A toxic combination of poor policies and programmes, unfair economic arrangements and bad governance may lead to unfavorable conditions. Ideally, the socio-politico-economic conditions in a society should be such that its citizens enjoy a favorable set of social resources, and that these resources are distributed fairly. The quality, quantity, and distribution of these resources, together, to a large extent, determine citizen's health and well-being. Opportunities to have an education, a healthy living environment, nutrition, healthcare and employment are some of those resources.

It is not difficult to see that all these social resources—which are known as social determinants of health—are shaped by public policies. For instance, the structure and quality of health care is considerably influenced by public policies made by the governments. Thus, public policy is a more fundamental determinant than the often-discussed social determinants of health. If we apply upstream and downstream analogy (4) then we see that most of our well-known social determinants of health such as education, employment, living environment exist in the midstream, and the public policy exists in the upstream. If we apply Geoffrey Rose's “causes of the causes (also referred to as the underlying causes)” concept (5) we can argue that these midstream resources and their quality are determined (or caused), to a large extent, by public policies. However, in most academic textbooks and journal articles on the social determinants of health, rarely is public policy covered, and often the mid-level factors are emphasized, which Raphael identifies as *mid-level focus* (6).

Graham argues that the social determinants of health concept has acquired a dual meaning referring both to the social factors promoting or undermining the health of individuals and populations as well as to the social processes underlying the unequal distribution of these factors between groups occupying unequal positions in society (7). Thus, the central concept of social determinants of health refers simultaneously to the determinants of health and to the determinants of inequalities in health (7). In other words, there are two dimensions of this concept—one is improvement in social factors that determine health and the other is equal distribution of those factors. As a result, the term “social determinants of health” is potentially confusing and may convey the message that it is all about the determining factors. This confusion may feed the policy assumption that health inequalities can be diminished by policies that focus only on the social determinants of health (8). There is now evidence that shows significant improvements in health determinants and consequently parallel improvements in population health may simultaneously increase the inequalities in determinants and health outcomes (9). The overall improvement may mask a persistent or even a growing inequality in the distribution of social determinants. Therefore, perhaps it is wise to slightly modify the term and make it something like “social determinants of health and related inequalities” so that it covers both *the determinants of health* and *the determinants of inequalities in health*.

Another source of ambiguity is the long and growing list of social determinants of health. Although initially a limited set of factors such as nutrition, education, employment, living environment were often emphasized, in recent times the list has grown substantially—both in peer reviewed literature and academic textbooks. In fact, it has grown so long that if someone wants to have a complete list of social determinants of health, his/her enthusiasm may quickly disappear after having realized how long the list is. Some of the most important social determinants of health that are dominant in the literature are education (10), housing and or living environment (11), income and its distribution (12), stress, early life, social exclusion, work, unemployment, social support, addiction, food, transport (13). In more recent literature, health system, gender, sexual orientation, social safety net (12), culture or social norms (14), media, stigma and discrimination (15), social capital (16), conflict, rule of law, racism, racialized legal status (17), immigration (18), family, religion (19), colonialism, and marginalization (20) have also been identified as social determinants of health. Strazdins et al. (21) identifies “time” as a social determinant of health, as healthy behavior, accessing health services, resting, and caring all require time. Also, the amount of time one can use for health-related activites is socially patterned and could therefore be a source of health inequalities. An article published in Iran identifies economic sanctions as a social determinant of health (22). American Medical Informatics Association believes that the access to broadband internet service should be added to the list of social determinants of health (23). There is also a growing subset of literature that examines social determinants of specific conditions such as depression, contraceptive use, and oral health.

As the Coordinator of a large subject—Social Determinants of Health—in which ~ 2000 students enroll each year, I receive questions from my students as to whether it is possible to have a complete list of social determinants of health. Some students perceive the list is too long, and suggest *nutrition, housing, education, employment, healthcare, and early life development* to classify as “elementary” social determinants of health and prioritize for policy interventions, as these determinants are likely to be important in most settings. In fact, WHO publication “the solid facts” (13) contains a concise list. However, rarely the academic textbooks follow this consistently. We also need to be mindful that the interactions between various determinants are not any less important (1).

Although there are reasons for variation in the list of social determinants of health, a long and varying list may have negative implications on our efforts in tackling them. For instance, a long list may give the impression that everything is social determinant of health, and therefore it is an unachievable task to tackle them. There might also be a question as to what degree of effort one should put on each determinant or which ones should be prioritized and on what basis. Objective data on the relative contribution of individual determinants on health and well-being are scarce and not always useful. Moreover, the contribution may vary across settings (24).

This long list of social determinants of health has implications on clinical practice and policy making. For instance, an overwhelming list could discourage physicians to consider screening social determinants of health. Already there are concerns about the requirement for and approaches to screening them and its benefits and unintended harms (25–27). A long list may discourage physicians to prioritize screening social determinants and referring patients to support services. Policy makers may also be less willing to go ahead with such a long and increasing list. In most government settings there are inherent barriers to adopt a social determinant approach in policy making (28). A long list may add further restrictions to adoption.

Unfortunately, there is no agreed taxonomy or criteria as to what should be considered a social determinant of health. In the literature, a subjective assessment—*whether social factors impacting health are avoidable through structural changes in policy and practice*—seems to be the dominant way of identifying a social determinant of health. Also, the word “social” remains ambiguous (29) and is difficult to define within a clear boundary. Although socioeconomic status is a major element which is often used to represent “social,” the meaning of “socioeconomic” may vary across settings. Perhaps this ambiguity and a growing list of determinants are two major reasons as to why the content list of most textbooks on social determinants of health vary significantly.

A clear understanding of the concept “social determinants of health” is critical for all major stakeholders including the public. However, there remains considerable ambiguity and confusion (2). For instance, in an interview with *NEJM Catalyst* the Chief Medical Officer of Health Leads stated:

“*A lot of why we're not doing better with the social determinants of health is because even though we know they're important, we don't really understand them*” (30).

Public understanding of the determinants of health is dominated broadly by biomedical and behavioral approaches, as is the coverage in the mainstream media. If ambiguity continues to exist, it might be difficult to communicate a clear message to the public. Raphael (31) argues that politicians tend to follow where the public goes, so helping the public to understand “social determinants of health” and consequently to demand change from governments is crucial. Currently policy-makers seldom solicit this concept to formulate public policy.

The past two decades have seen a resurgence of international interest in the non-medical and non-behavioral precursors of health and illness, and social determinants of health are at the center of this focus. As Marmot says, “social determinants of health” has become part of the language in some settings (32). Now an unprecedented opportunity exists to improve health on a global scale—particularly for underprivileged subgroups of the community. We should endeavor to eliminate the existing ambiguities and confusions and let this current momentum of social determinants of health guide our public policy.

## Author Contributions

MI conceived the idea of writing this opinion piece and wrote the manuscript.

### Conflict of Interest Statement

The author declares that the research was conducted in the absence of any commercial or financial relationships that could be construed as a potential conflict of interest.
